# Endoscopic Full‐Thickness Resection for Gastrointestinal Subepithelial and Epithelial Neoplasia: Current Status, Indications, and Technical and Oncological Challenges—A Multispecialty Perspective

**DOI:** 10.1111/den.70221

**Published:** 2026-07-09

**Authors:** Seiichiro Abe, Kenichiro Imai, Teppei Akimoto, Takashi Kanesaka, Yosuke Minoda, Noriko Nishiyama, Chiko Sato, Akira Ouchi, Hon Chi Yip, Takao Itoi, Mitsuhiro Fujishiro, Yutaka Saito

**Affiliations:** ^1^ Endoscopy Division National Cancer Center Hospital Tokyo Japan; ^2^ Division of Endoscopy Shizuoka Cancer Center Shizuoka Japan; ^3^ Division of Research and Development for Minimally Invasive Treatment Cancer Center, Keio University School of Medicine Tokyo Japan; ^4^ Department of Gastrointestinal Oncology Osaka International Cancer Institute Osaka Japan; ^5^ Department of Medicine and Bioregulatory Science, Graduate School of Medical Sciences Kyushu University Fukuoka Japan; ^6^ Departments of Gastroenterology and Neurology, Faculty of Medicine Kagawa University Kagawa Japan; ^7^ Division of Endoscopy Yokohama City University Medical Center Yokohama Japan; ^8^ Department of Gastroenterological Surgery Aichi Cancer Center Hospital Nagoya Japan; ^9^ Department of Surgery The Chinese University of Hong Kong Hong Kong Hong Kong; ^10^ Department of Gastroenterology and Hepatology Tokyo Medical University Hospital Tokyo Japan; ^11^ Department of Gastroenterology, Graduate School of Medicine The University of Tokyo Tokyo Japan

**Keywords:** endoscopic full‐thickness resection, epithelial neoplasms, minimally invasive surgical procedures, subepithelial neoplasms, wound closure techniques

## Abstract

Endoscopic full‐thickness resection (EFTR) has emerged as a minimally invasive treatment for small gastrointestinal subepithelial neoplasms (SETs). In particular, the development of novel closure methods such as clip‐line and various endoscopic suturing techniques has enabled the secure closure of any resultant defects. During the Advanced Therapeutic Endoscopy session at the Endoscopic Forum Japan 2025, we summarized the current status of EFTR for SETs and discussed future clinical applications for epithelial neoplasms from oncological and technical perspectives. Although predominantly documented in gastric subepithelial neoplasms, EFTR is also used to treat select esophageal, duodenal, and colorectal SETs. While extensively evaluated for gastric gastrointestinal stromal tumors (GISTs), relatively low R0 resection rates have limited its broader clinical adoption; however, the novel no‐touch EFTR technique could overcome this challenge and facilitate the adoption of this procedure for small gastric GISTs. Applying EFTR to epithelial tumors requires strict assessment of oncological clearance, given the risks of lymph node metastasis and peritoneal seeding following full‐thickness breaches. At the end of the session, we voted on the appropriateness of EFTR for epithelial neoplasms across different organs, balancing oncological risks against technical feasibility. All nine participants (100%) supported EFTR for rectal lesions, and eight (89%) supported its use for gastric lesions. Future investigations and technical innovations are required to expand the clinical indications for EFTR in epithelial neoplasms.

## Graphical abstract

In this review article, we summarized the current status of EFTR for SETs and discussed future clinical applications for epithelial neoplasms from oncological and technical perspectives. EFTR is clinically feasible for treating gastric SETs. Future investigations and technical innovations are required to expand the clinical indications for EFTR in epithelial neoplasms, particularly for rectal and gastric lesions.

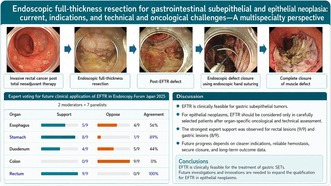

## 1 Introduction

Endoscopic full‐thickness resection (EFTR) has emerged as a minimally invasive treatment for gastrointestinal (GI) subepithelial tumors (SETs), which are often difficult to remove using conventional endoscopic techniques. Furthermore, this procedure has proven feasible for transluminal organ‐preserving resection with secure defect closure [1, 2].

Although laparoscopic and robot‐assisted surgeries with lymph node dissection have been standardized for the radical resection of epithelial neoplasms, critical drawbacks remain, including procedure‐related morbidity, prolonged operative times and hospitalization, increased medical costs, and irreversible functional impairment following partial or total organ resection [3, 4]. Consequently, clinicians have reconsidered the parameters for local epithelial neoplasm resection, expanding the indications and curability assessments for both endoscopic mucosal resection (EMR) and endoscopic submucosal dissection (ESD) [5, 6]. These minimally invasive alternatives are particularly relevant for older patients or those with significant comorbidities. Moreover, they are crucial for treating lesions in anatomically or functionally critical segments, where standard surgical intervention imposes profound physiological and quality‐of‐life burdens.

Therefore, EFTR serves as an alternative treatment for select invasive cancers where adequate deep resection margins cannot be achieved via EMR or ESD. However, robust clinical evidence supporting EFTR for epithelial neoplasms remains limited, and its clinical indications and technical feasibility are not yet clearly established. In this review, we summarize the current status of EFTR for SETs and discuss future clinical applications for epithelial neoplasms from oncological and technical perspectives.

## 2 Methods

During the Advanced Therapeutic Endoscopy session at the Endoscopic Forum Japan 2025, two moderators and seven panelists presented an overview of EFTR for SETs and discussed current oncological and technical challenges regarding its application in epithelial neoplasms. Each panelist presented a narrative review on a specific EFTR topic at the March 1, 2025 meeting in Tokyo. The session concluded with informal voting on the appropriateness of EFTR for specific organs.

## 3 Findings

### 3.1 Categorization of Endoscopic Resection Techniques

EFTR is categorized into exposed and nonexposed techniques [7, 8]. Exposed EFTR involves full‐thickness resection followed by defect closure. Nonexposed EFTR uses a “close and resect” approach, approximating the layers deep to the lesion prior to full‐thickness resection to prevent perforation. Device‐assisted resection, a subset of nonexposed EFTR, is typically performed using an over‐the‐scope clip (OTSC, Ovesco Endoscopy, Tuebingen, Germany), a full‐thickness resection device (FTRD, Ovesco Endoscopy, Tuebingen, Germany), or a tip‐mounted ligation device. The FTRD is a specialized device designed for one‐step full‐thickness resection, incorporating an OTSC mounted over a cap and an integrated snare system. Submucosal tunneling endoscopic techniques, such as peroral endoscopic tumor resection (POET) and submucosal tunneling endoscopic resection (STER), can be categorized as either exposed or nonexposed EFTR depending on the specific approach [7, 8, 9]. However, these methods generally fall under the “third‐space endoscopy” category because they derive from peroral endoscopic myotomy and utilize a mucosal flap valve mechanism [8].

Additionally, related procedures such as endoscopic muscular dissection (EMD), peranal endoscopic myectomy (PAEM), endoscopic intermuscular dissection (EID), and endoscopic subserosal dissection (ESSD) have been reported [10, 11, 12, 13, 14]. By preserving the integrity of the muscularis propria or serosa at the defect site, these approaches theoretically reduce the risks of luminal collapse, impaired visualization, and peritonitis compared with EFTR. Conversely, they present limitations regarding deep margin assessment and true R0 resection. Furthermore, the resection depth is primarily dictated by the target lesion's location and remains difficult to control intraoperatively.

### 3.2 Status of EFTR in SETs

**FIGURE 1:**
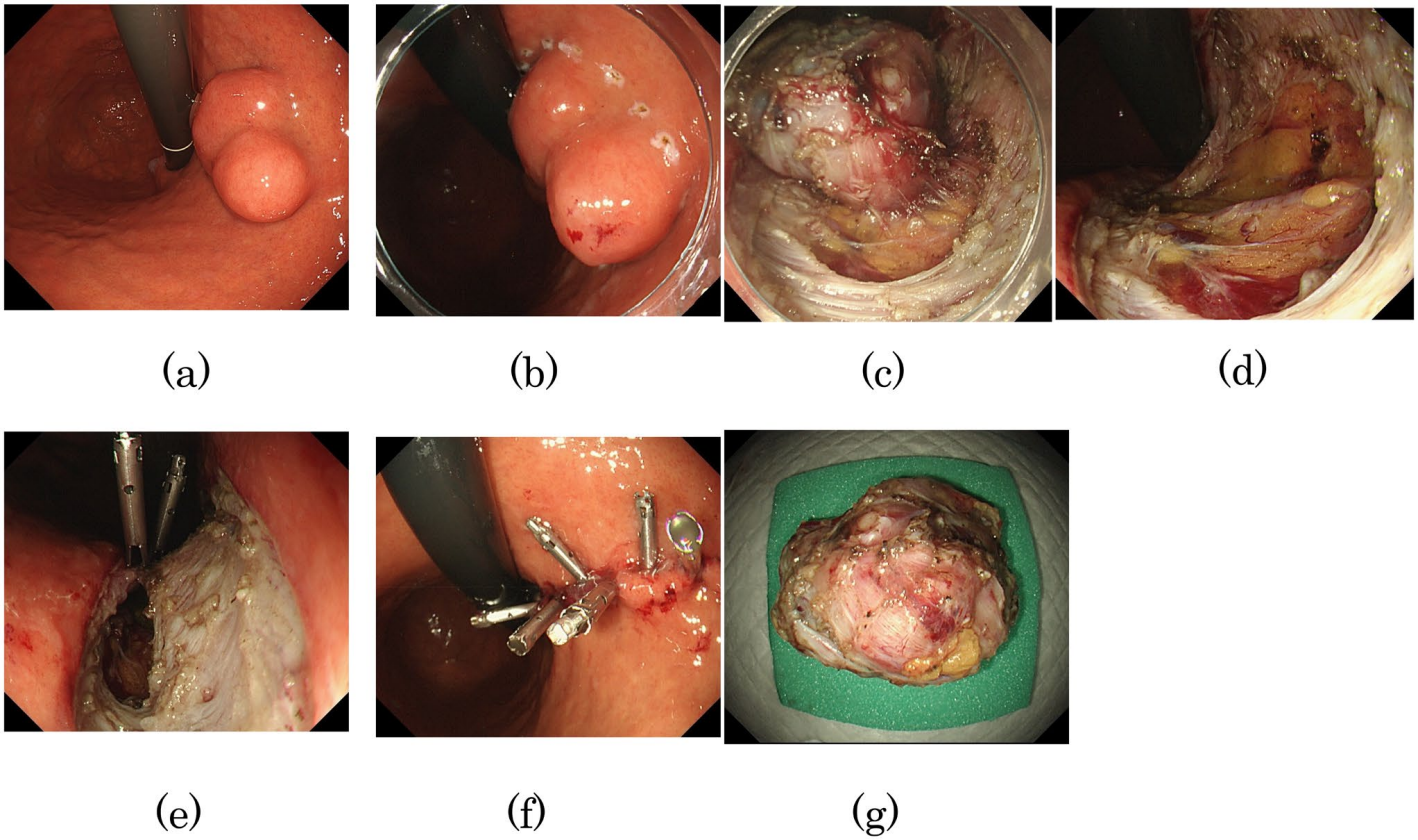
Endoscopic full‐thickness resection of gastric subepithelial tumors (SETs). (a) White‐light endoscopy revealed a SET in the lesser curvature of the upper gastric body. (b) Circumferential markings were placed around the lesion. (c) The muscular layer below the lesion was dissected. (d) A defect remained following full‐thickness resection. The specimen was resected en bloc. (e) The muscular layer was closed using endoclips. (f) Complete mucosal closure was achieved. (g) The resected specimen.

Suzuki and Ikeda [15] first introduced the concept of EFTR in 2001, and Zhou et al. [16] subsequently demonstrated its feasibility and safety for gastric SETs in 2011. Since then, numerous studies—predominantly from China—have reported using EFTR to resect small SETs. EFTR for gastric gastrointestinal stromal tumors (GISTs) is considered most suitable for select intraluminal growth‐type lesions, whereas extraluminal or mixed‐growth tumors require careful multidisciplinary assessment to ensure procedural safety and secure free lateral margins; thus, these complex tumors may be better managed surgically. Since 2020, Japan has permitted gastric EFTR for intraluminally growing, non‐ulcerated gastric SETs measuring 11–30 mm—primarily confirmed or suspected GISTs (Figure 1)—under the Advanced Medical Care Type A framework [17]. This regulatory framework allows the clinical implementation of innovative procedures with established safety and feasibility profiles, partially covered by Japan's National Health Insurance System. A Japanese multicenter prospective study evaluating gastric EFTR outcomes in 46 patients with SETs ≤ 30 mm reported a 100% en bloc resection rate and a 77% R0 resection rate, with no cases requiring surgical conversion [17].

The necessity of R0 resection in EFTR warrants further investigation, as a previous report suggested that microscopically positive margins (R1/RX) following endoscopic GIST resection do not significantly increase recurrence risk [18]. Consequently, Japanese and European guidelines do not strongly recommend routine repeat surgery for patients with macroscopically negative but histologically positive margins [19, 20]. Nevertheless, achieving R0 resection remains the optimal clinical outcome. The no‐touch EFTR approach, wherein resection includes a 5–10 mm perimeter outside the tumor margins, reportedly achieves a 100% R0 resection rate [21].

### 3.3 Organ‐Specific EFTR in SET

Table 1 summarizes the clinical indications, adverse event risks, and R0 resection rates of EFTR for GI SETs by organ.

**TABLE 1:** Clinical indications and approach for EFTR of subepithelial neoplasms.

	Exposed‐EFTR		Nonexposed EFTR
	Non‐tunneled EFTR	Tunneled EFTR	FTRD, OTSC
Location	Stomach	Esophagus, Cardia	Colon, Duodenum
Lesion size	< 3 cm	< 3 cm	< 2 cm
Risk of pneumoperitoneum	High	Mod	Low
Risk of dissemination risk of tumor cells due to capsule injury	Mod	Mod	Low
R0 resection rate	High	Limited	Limited

Abbreviations: EFTR, endoscopic full thickness‐resection; FTRD, full thickness resection device; OTSC, over‐the‐cope clip.

#### 3.3.1 Esophagus

Because STER and POET are considered the gold standard for muscularis propria‐derived SETs < 40 mm, the indications for esophageal EFTR remain limited [22, 23, 24]. A large series of STER and POET procedures for upper GI lesions (*n* = 180; 90% esophageal) identified tumor size ≥ 30 mm and irregular shape as risk factors for piecemeal resection and postoperative complications [25]. Similarly, Onimaru et al. [24] reported that tumors with a minor axis > 30 mm or a tumor mass index (TMI) > 1000 (major axis diameter [mm] × minor axis diameter [mm]) should be treated surgically rather than endoscopically. Furthermore, multiple studies reported no recurrence over median follow‐up periods exceeding 36 months [24, 25].

#### 3.3.2 Stomach

A systematic review of 19 studies evaluating EFTR for gastric SETs reported complete resection and surgical conversion rates of 99.3% and 0.09%, respectively [26]. A 2023 literature review summarizing 27 studies demonstrated an en bloc resection rate of 98.4%, an R0 resection rate of 96.5%, and a surgical conversion rate of 0.56%, indicating consistently favorable outcomes [27]. Comparative analyses of various closure techniques, including clips, purse‐string suturing (PSS), and the OTSC, showed no significant differences regarding complications or surgical conversion rates [28, 29]. The reopenable‐clip over the line method (ROLM) proposed by Nomura et al. [30] was associated with reduced postoperative pain, fasting times, and hospitalization lengths compared with PSS [31]. Several retrospective studies demonstrated favorable long‐term outcomes for the endoscopic resection of gastric GISTs, including via ESD, STER, and EFTR [32, 33, 34]. Notably, Gao et al. [34] evaluated 460 very low‐risk and 72 low‐risk patients, reporting 5‐year overall survival rates of 99.7% and 100%, respectively.

#### 3.3.3 Duodenum

Exposed duodenal EFTR, which requires maneuvering within a narrow lumen, is challenging due to the difficulty of closing large defects and the high morbidity associated with perforation‐related leakage. Consequently, nonexposed EFTR is generally preferred. OTSC‐assisted duodenal EFTR has proven feasible and safe [35]. An international multicenter analysis demonstrated the clinical utility of device‐assisted EFTR for duodenal neuroendocrine tumors measuring approximately 10 mm, reporting technical success and R0 resection rates of 95.9% and 71.9%, respectively. The R0 resection rate for lesions in the proximal third of the duodenal bulb was significantly lower than for those in the distal duodenum (62.0% vs. 83.9%, respectively). Severe adverse events occurred in three patients (1.8%) [36].

#### 3.3.4 Colon and Rectum

EFTR using the FTRD is standard practice in Western countries to ensure full‐thickness closure prior to resection. A multicenter trial reported technical success and R0 resection rates of 95.5% and 87.0%, respectively, among patients with colorectal SETs. Additionally, R0 resection rates were higher for lesions < 20 mm. Adverse events occurred in 9.9% of cases, necessitating emergency surgery in 2.2% [37]. Most studies evaluating EFTR excluded lesions > 30 mm, as the ability to aspirate lesions into a 21‐mm‐diameter cap depends on tissue characteristics such as scarring, rigidity, and mobility [38].

### 3.4 EFTR In Epithelial Tumors—Advantages and Disadvantages Compared With Surgical Resection

**FIGURE 2:**
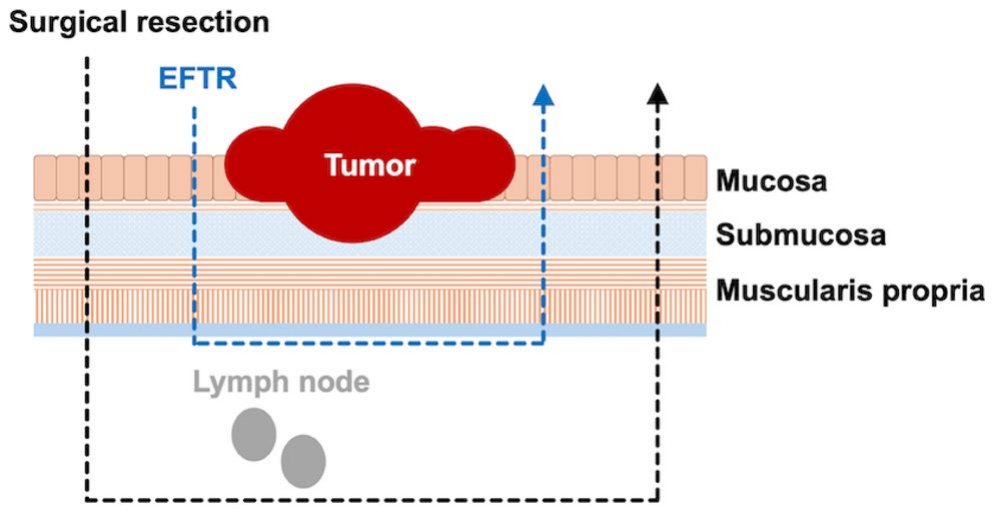
Scheme of endoscopic full‐thickness resection of epithelial neoplasms.

The principal oncological limitation of EFTR, compared with conventional surgical resection, is its inability to include lymph node dissection (Figure 2). Clinically, EFTR and surgical resection differ regarding procedural invasiveness, hospitalization lengths, peritoneal seeding risks, and technical challenges in achieving hemostasis (Table 2). Surgical procedures are highly invasive and require prolonged postoperative recovery, whereas EFTR offers substantial advantages through reduced invasiveness and faster recovery times. Another practical limitation of EFTR is that the resected specimen must be retrieved transorally or transanally, inherently restricting maximum removable lesion size. Furthermore, most published EFTR studies focus on SETs, resulting in limited evidence regarding its safety and feasibility for epithelial tumors [17, 39, 40].

**TABLE 2:** Clinical and procedural characteristics of EFTR compared with surgical resection.

Procedural invasiveness	Less invasive than surgery; limited by the requirement to retrieve the specimen orally or transanally
2 Length of hospitalization	Shorter than surgery
3 Risk of peritoneal seeding	Potentially higher than surgery
4 Technical difficulty of hemostasis	More challenging than surgery

Abbreviation: EFTR, endoscopic full‐thickness resection.

Beyond procedural invasiveness, EFTR poses unique clinical risks compared with surgery. Unlike surgical resection, EFTR carries a risk of peritoneal seeding; therefore, clinicians must not only establish strategies to minimize this risk but also clarify its true impact on tumor recurrence. Hemostasis presents greater technical challenges during EFTR, particularly for extraluminal bleeding, because current endoscopic devices offer less effective hemostatic capabilities than surgical instruments. Accordingly, the standardization and wider adoption of EFTR necessitate further advances in endoscopic hemostasis technology, including the development of dedicated devices.

### 3.5 Clinical Indications for EFTR in Epithelial Tumors From an Oncological Perspective

SETs generally present a lower risk of lymph node metastasis (LNM) than epithelial tumors; therefore, EFTR provides definitive local treatment for select SETs, as R0 resection alone typically ensures oncological clearance. In contrast, epithelial tumors present a greater challenge, as peritoneal dissemination and tumor recurrence from occult LNM cannot be entirely excluded following EFTR. Therefore, EFTR should be limited to patients for whom long‐term disease control can be achieved via local resection alone. EFTR may also serve as an alternative when R0 resection via ESD is unlikely, such as for lesions with severe fibrosis. Consequently, the oncological indications for EFTR must be evaluated in an organ‐specific manner, balancing biological risks against technical feasibility.

#### 3.5.1 Esophagus

Residual or recurrent lesions with severe fibrosis following chemoradiotherapy (CRT) may benefit from EFTR if R0 resection is achievable; however, T1b esophageal cancers frequently exhibit higher LNM rates (20.1%–28.6%) [41, 42]. As such, EFTR is not considered an appropriate treatment for typical T1b disease.

#### 3.5.2 Stomach

EFTR may be a suitable approach for invasive submucosal cancers and ulcerated gastric lesions, as it enables R0 resection without damaging the lesion itself. The LNM rate for T1b gastric cancers without lymphovascular invasion is ≤ 3% [43, 44], making these cases viable candidates for EFTR. Histologically, undifferentiated‐type early gastric cancer (EGC) carries a higher LNM risk and greater likelihood of positive horizontal margins than differentiated‐type EGC [45, 46]; therefore, EFTR should primarily be limited to differentiated‐type EGC.

#### 3.5.3 Duodenum

Nonexposed EFTR may be indicated for recurrent intramucosal carcinomas or lesions with severe fibrosis that are difficult to treat via conventional EMR/ESD [35, 47]. Because T1b duodenal cancers exhibit relatively high LNM rates (14%–22%) [48, 49], surgical resection remains the treatment standard. EFTR may be considered for neuroendocrine tumors (NETs), which carry a higher risk of deep‐margin positivity via EMR or ESD, given that endoscopic treatment is recommended for NETs ≤ 10 mm [50].

#### 3.5.4 Colon and Rectum

T1b colorectal cancers without lymphovascular invasion have low LNM rates (1.2%–1.9%) [51, 52, 53], making EFTR a viable option for select patients. Rectal surgery is highly invasive and may require a temporary or permanent ostomy, severely impacting patient quality of life; therefore, EFTR may be preferred in these scenarios. EFTR may also be appropriate for residual lesions following CRT or total neoadjuvant therapy, and for anatomically challenging lesions, such as those involving the appendiceal orifice or extending into diverticula. PAEM, also referred to as EID, enables dissection beyond the submucosal layer [13, 14]. Developed as an extension of ESD to overcome technical challenges like severe fibrosis and muscle traction, it enables dissection within the intermuscular plane while preserving the outer longitudinal muscle. This technique is primarily applied to lower rectal lesions. While these intermuscular approaches offer technical advantages—including reduced luminal collapse and improved endoscopic visualization—they face limitations regarding accurate deep resection margin assessment and ensuring R0 resection for T2 cancers.

### 3.6 Clinical Indications for EFTR in Epithelial Tumors From a Technical Perspective

**FIGURE 3:**
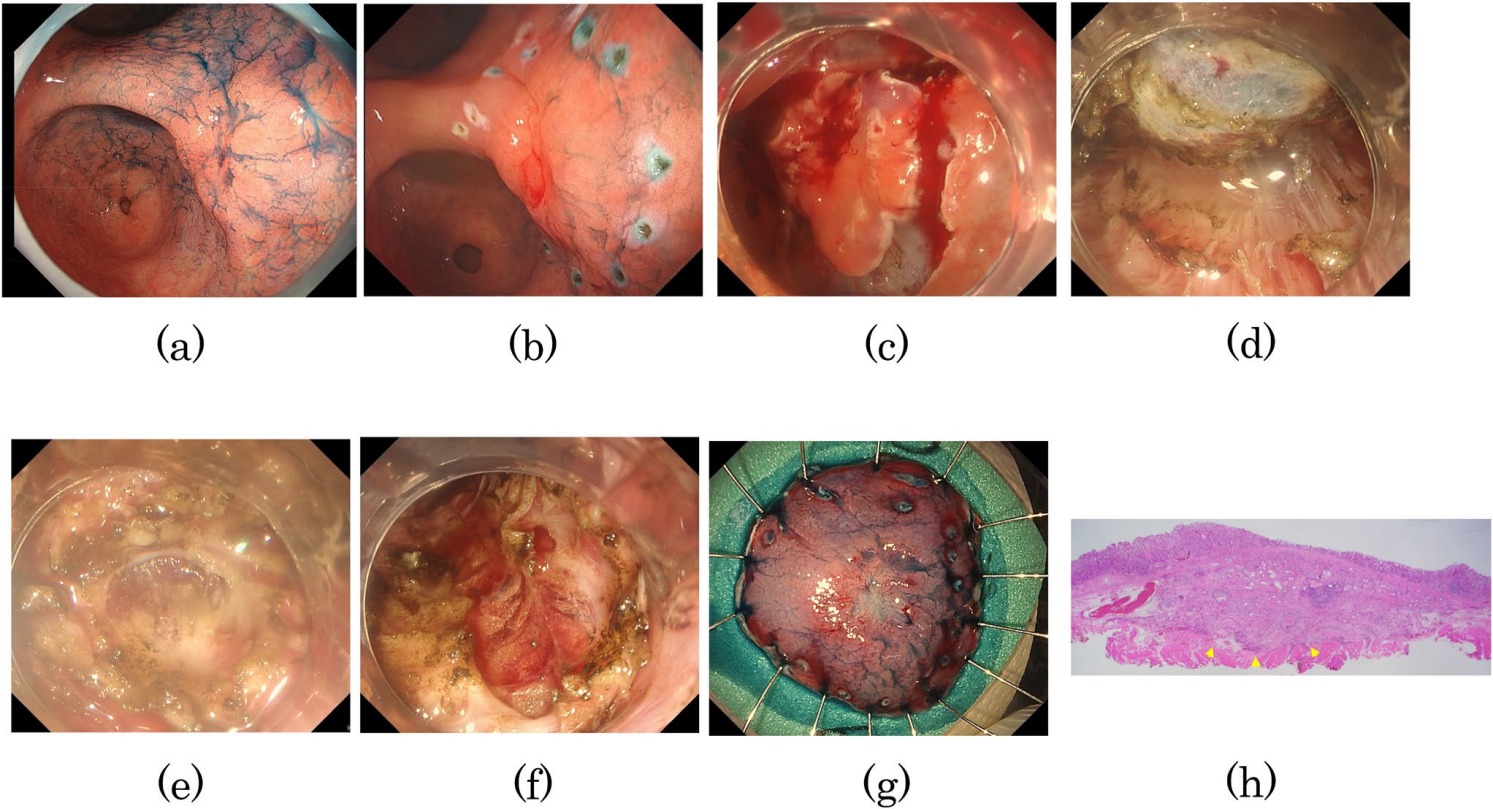
Endoscopic full‐thickness resection of invasive gastric cancer. (a) Endoscopy identified a gastric cancer with deep submucosal invasion on the posterior wall of the gastric incisura. (b) Circumferential markings were placed around the lesion. (c) A circumferential mucosal incision was performed. (d) Whitish tissue was observed invading the muscular layer. (e) The muscular tissue was dissected at deeper sites. (f) A defect remained following full‐thickness resection. (g) The specimen was resected en bloc. (h) Histopathological analysis confirmed advanced gastric cancer invading the muscularis propria with a negative vertical margin.

**FIGURE 4:**
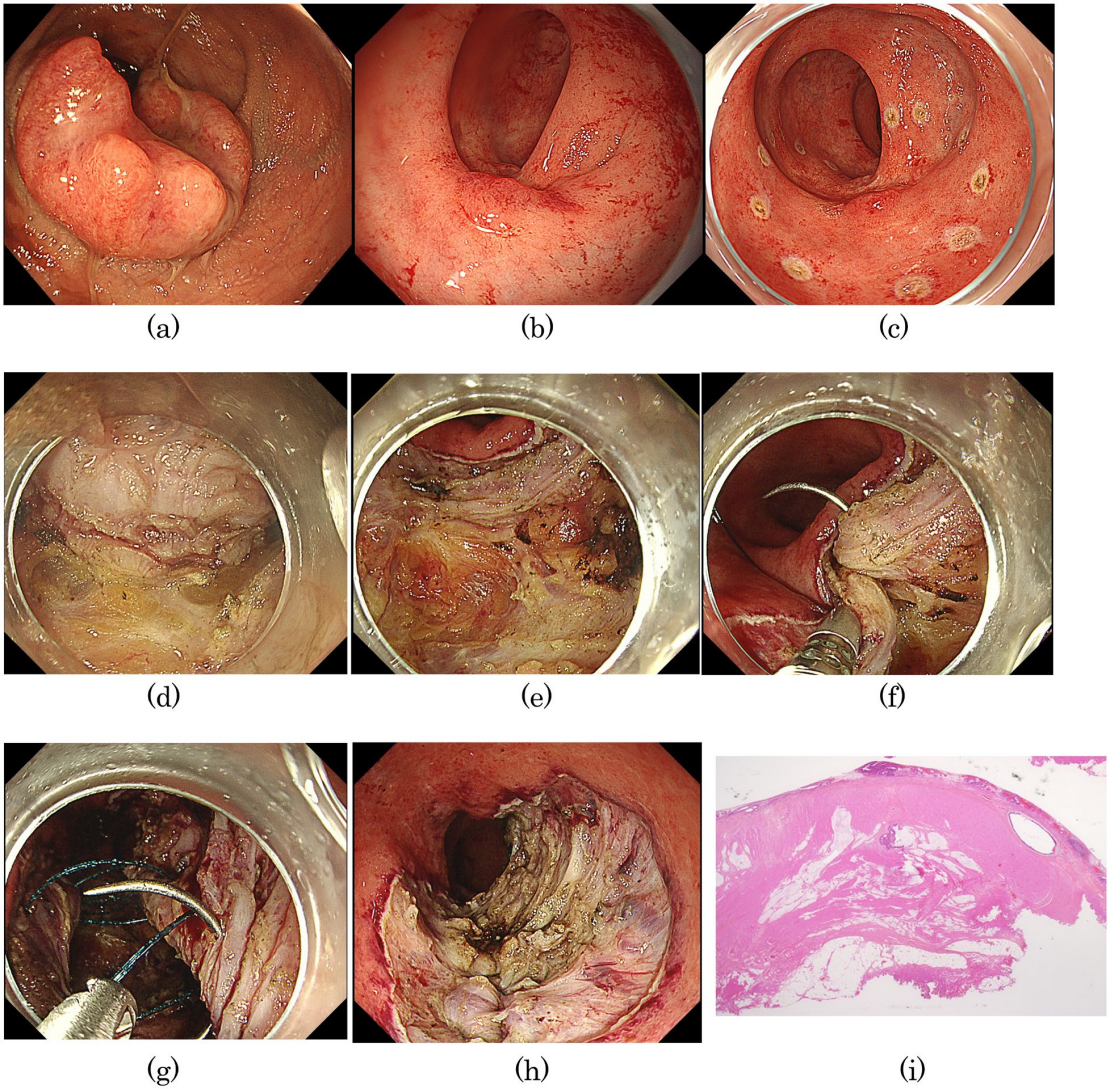
Endoscopic full‐thickness resection (EFTR) of locally advanced rectal cancer following total neoadjuvant therapy (TNT). (a) Initial endoscopy revealed a type 2 rectal tumor in the lower rectum measuring 40 mm, staged as cT3N1aM0. TNT—consisting of short‐course radiotherapy (25 Gy in 5 fractions) and six cycles of CAPOXIRI (capecitabine, oxaliplatin, and irinotecan)—was administered. (b) Six months following TNT, follow‐up endoscopy showed scar formation with a small erythematous nodule at the lesion's center; a biopsy revealed adenocarcinoma. (c) The lesion was diagnosed as localized residual disease confined to the mucosa, and EFTR was performed following circumferential marking. (d) A circumferential incision and submucosal dissection were initially performed, followed by full‐thickness resection of the muscularis propria at the scarred area suspected of harboring residual tumor. (e) The post‐EFTR defect exhibited preserved muscularis propria at the margins, whereas the central area demonstrated adventitial exposure. (f) The area with exposed adventitia was closed using the endoscopic hand suturing (EHS) technique. (g) Muscular defect closure was achieved by approximating the muscular layers via a V‐Loc suture. (h) The muscular defect was further approximated by tightening the suture. (i) Histopathology revealed residual mucosal tumor alongside residual tumor cells with mucin pools extending from the muscularis propria to the adventitia.

EFTR comprises four key procedural steps: full‐thickness resection, hemostasis, closure, and specimen retrieval. Technically, EFTR presents multiple challenges across these steps, and its feasibility varies significantly depending on the anatomical location of the tumor [54]. Although EFTR is generally considered technically feasible in the stomach, its execution in other areas proves considerably more challenging. Esophageal EFTR poses significant risks due to the proximity of vital adjacent structures, such as the bronchi, major blood vessels, and nerves. The duodenum is also a particularly challenging site due to the risk of pancreatic injury and subsequent peritonitis. Secure duodenal defect closure is crucial, as bile or pancreatic juice leakage can result in severe, life‐threatening complications. In contrast, the extraperitoneal anatomy of the lower rectum reduces the risk of peritonitis or dissemination, and its wider lumen facilitates more reliable suturing and defect closure. Despite these challenges, EFTR remains a promising treatment for various GI epithelial tumors (Figures 3 and 4); the specific technical hurdles are detailed below.

#### 3.6.1 Full‐thickness Resection—Compromised Visibility Post‐Resection

The loss of luminal insufflation following full‐thickness GI wall transection markedly impairs anatomical visualization [55]. In the stomach, mesenteric or serosal coverings may help maintain pneumoperitoneum; however, evidence of this phenomenon in other organs remains limited. This compromised visibility prolongs the procedure, necessitating either measures to prevent decompression or proficiency in operating within decompressed environments. Traction‐assisted full‐thickness resection using clip‐line traction offers one solution for identifying both incision lines and tissue dissection planes during muscle transection in poorly visualized fields [56].

#### 3.6.2 Hemostasis

Managing intraoperative and delayed bleeding into the peritoneal cavity remains a critical concern during EFTR [57], as reliable endoscopic hemostatic techniques are lacking. Although studies on EFTR for gastric GISTs provide insights into intraoperative safety management [58], angiographic intervention remains the only treatment for delayed extraluminal bleeding.

#### 3.6.3 Wound Closure

No definitive closure method has been established for EFTR due to limited comparative data. Although endoloop closure is a common technique, newer suturing devices may offer greater reliability [56, 59, 60]. In general, the hierarchy of closure strength is as follows: TTS clips< OTSC < needle‐and‐suture systems [61, 62]. While most current methods achieve lateral closure, future development must focus on systems enabling directional control of the suture line. We summarize the characteristics of full‐thickness closure techniques by device type below.

##### 3.6.3.1 String clip suturing method

**FIGURE 5:**
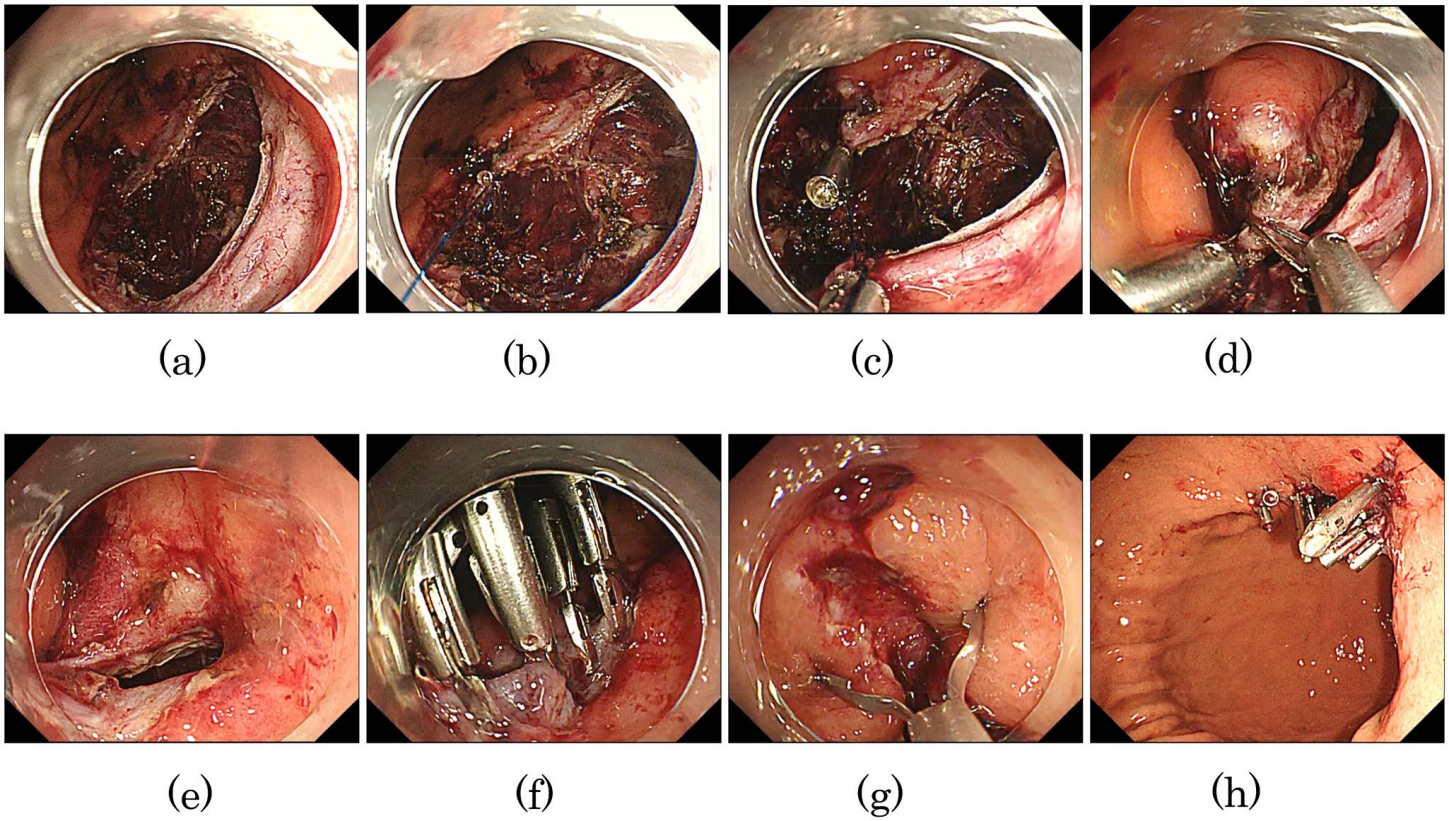
String‐clip suturing method. (a) A full‐thickness defect was observed on the lesser curvature of the gastric body following endoscopic full‐thickness resection. (b) A clip with an attached suture was advanced through the working channel and deployed on the muscular layer's edge. (c) The suture and the contralateral muscular edge were grasped with a second clip, and traction was applied to approximate the defect edges. (d) The approximated muscular layers were secured using an additional clip introduced through the opposite working channel of a dual‐channel gastroscope. (e, f) After cutting the suture, the remaining muscular gap was closed by inverting the muscular layer. (g) Following muscular layer closure, mucosa‐to‐mucosa approximation was performed. (h) Complete defect closure was achieved.

TTS clips alone rarely achieve sufficient inversion or durable full‐thickness closure; therefore, their use is restricted to very small defects. Furthermore, their efficacy is highly operator‐dependent [63]. Accordingly, clinicians often combine TTS clips with the string‐clip method, wherein a preloaded suture clip provides directed traction to approximate the defect and facilitate clip placement [64, 65]. This concept has been adopted into other full‐thickness closure techniques, such as ROLM and line‐assisted complete closure [30, 66]. Although these techniques utilize inexpensive and widely available devices, they require substantial technical expertise to ensure reliable closure (Figure 5).

##### 3.6.3.2 OTSC‐Based Full‐Thickness Closure

**FIGURE 6:**
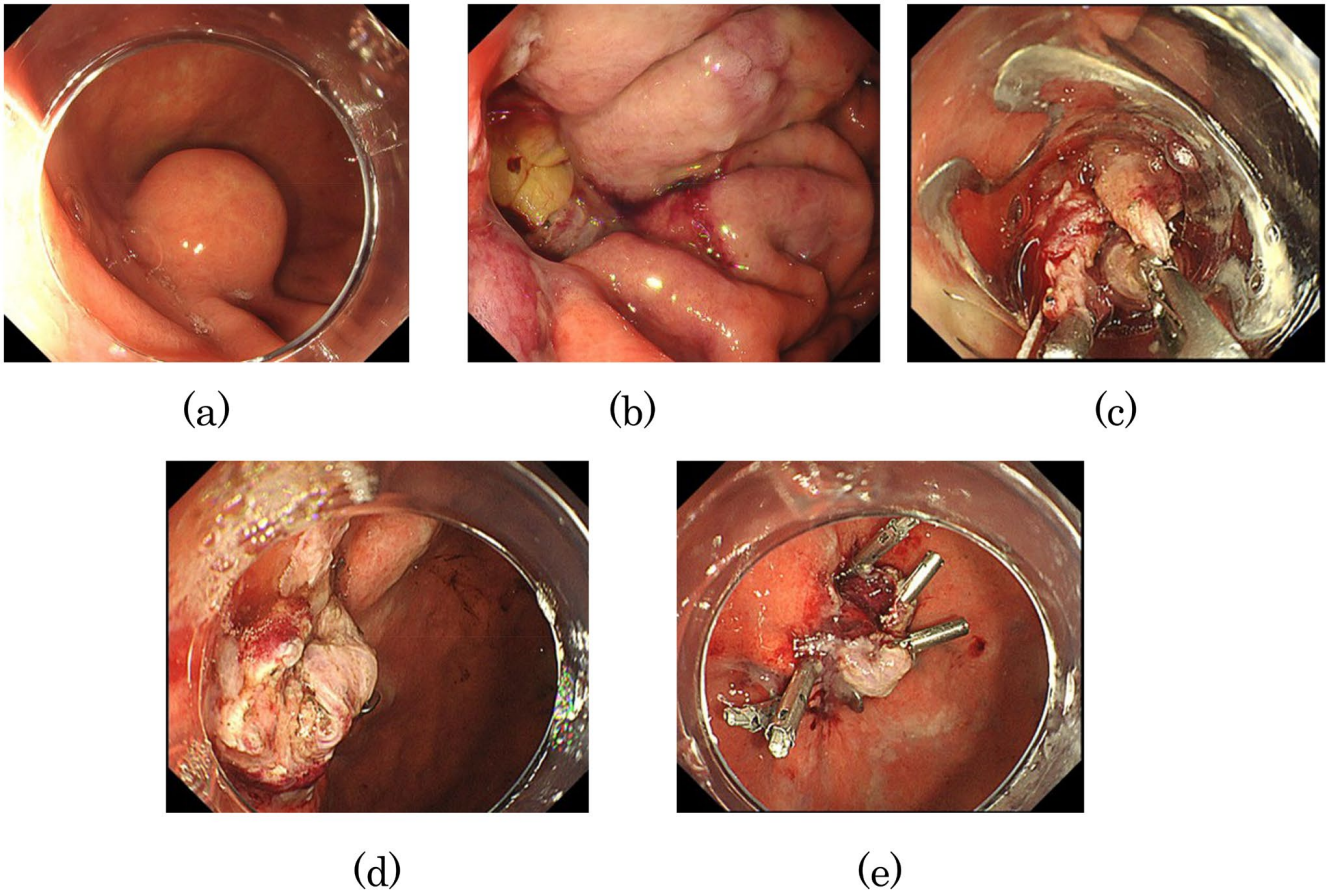
Endoscopic defect closure using the over‐the‐scope clip (OTSC) system. (a) White‐light endoscopy revealed a subepithelial tumor in the greater curvature of the gastric fundus. (b) A 15‐mm full‐thickness defect remained following standard endoscopic full‐thickness resection. (c) Two small‐caliber grasping forceps were advanced through the dual working channels of a gastroscope mounted with an OTSC (10 mm, gc‐type), grasping the seromuscular layers on both sides of the defect. (d) Under adequate suction, the grasped tissue was drawn into the OTSC cap, and the clip was deployed. (e) Complete closure was achieved using standard through‐the‐scope clips.

OTSC systems offer two major advantages for full‐thickness defect closure: (1) reliable tissue inversion and closure and (2) highly standardized procedural steps. However, system limitations include the requirement for additional grasping devices to pull tissue into the cap, the necessity of multiple clips for larger defects (increasing costs), and the potential need for reinforcements to prevent leakage between clip gaps [29, 67, 68] (Figure 6).

##### 3.6.3.3 Dedicated Needle‐and‐Suture Devices

**FIGURE 7:**
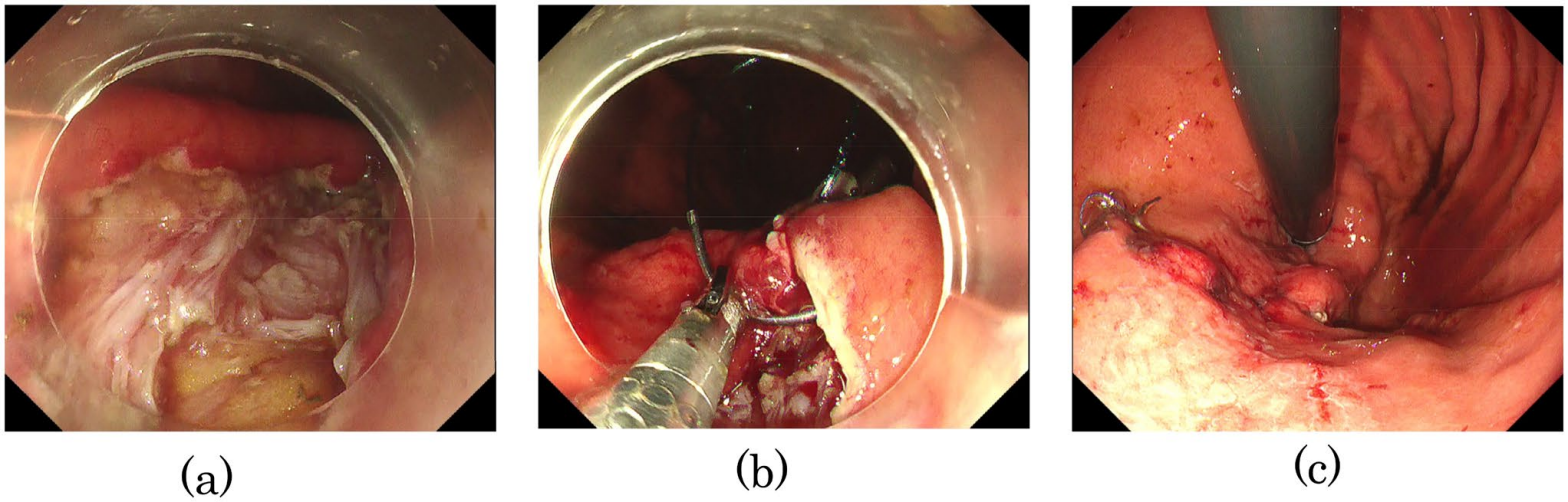
Endoscopic hand suturing (EHS) for defect closure. (a) A full‐thickness defect remained at the posterior cardia following endoscopic full‐thickness resection of a gastric gastrointestinal stromal tumor. (b) EHS was applied to close the defect using a suturing technique with the SutuArt device and a 3–0 V‐Loc suture. Both the mucosal and muscular layers were sutured as a single layer. (c) The final view demonstrated complete closure achieved via EHS.

Specialized suturing platforms—such as OverStitch (Apollo Endosurgery Inc., Austin, TX, USA), Zeosuture M (ZEON Medical Inc., Tokyo, Japan), and SutuArt (Olympus Medical Systems Corp., Tokyo, Japan) enable wound closure that closely resembles surgical suturing, providing robust full‐thickness apposition [60, 69]. Despite their strengths, these devices face limitations including bulky platforms, restricted maneuverability, and higher costs that hinder their widespread clinical adoption (Figure 7).

#### 3.6.4 Post‐Procedural Stricture—A Limitation on Lesion Size

The risk of postprocedural stricture formation at the wound closure site directly limits the maximum resectable lesion size. To overcome this limitation, however, a hybrid technique combining EFTR and ESD may be feasible. This approach, which involves full‐thickness resection of the deeply invasive tumor portion while preserving the muscularis propria beneath the surrounding normal mucosa, currently remains purely conceptual.

#### 3.6.5 Specimen Retrieval—A Limitation On Lesion Size

Transoral retrieval techniques inherently limit specimen size; for gastric GISTs, the maximum retrievable diameter is approximately 30 mm [17]. However, because epithelial tumors are more pliable than GISTs, they may permit the removal of larger lesions. Although resected muscle layer thickness influences retrieval feasibility, the risk of postprocedural stricture likely remains the primary factor defining maximum resectable lesion size.

In summary, while EFTR for epithelial neoplasms holds substantial promise as a curative option for select GI lesions, its clinical application remains limited, primarily due to technical challenges and anatomical constraints. Ensuring secure, robust wound closure is essential not only to prevent adverse events but also to minimize the oncological risk of peritoneal dissemination. Future innovations in dedicated wound closure and hemostasis technologies are therefore essential to standardize this procedure.

### 3.7 Technical Challenges of Endoscopic Wound Closure for EFTR

Full‐thickness wound closure techniques are classified into three major types: layer‐to‐layer, inversion, and eversion suturing [57]. Durable closure is essential to prevent leakage and postoperative fistula formation. Among these techniques, layer‐to‐layer apposition is theoretically ideal for promoting wound healing; however, achieving this configuration endoscopically remains technically challenging. Eversion closure is generally discouraged, as it predisposes patients to leakage. Therefore, inversion closure represents the most practical and safe approach to full‐thickness defect closure, given the capabilities of current devices.

#### 3.7.1 Organ‐Specific Considerations

The narrow esophageal lumen poses a substantial risk of postoperative stricture if excessive tissue approximation occurs. Although the spacious gastric lumen permits a broad range of device options, the organ is unfixed and prone to eversion during transection; therefore, reliable inversion requires dedicated techniques and devices. The confined and sharply angulated duodenal lumen, combined with continuous exposure to bile and pancreatic juice, necessitates highly precise and secure closure. In the colon, particularly the proximal segments, scope reinsertion can be challenging, making TTS‐based approaches more suitable. Finally, because the rectum is retroperitoneally fixed, additional methods are often required to achieve adequate defect approximation.

#### 3.7.2 Nonexposed Approach

Following perforation, the bowel wall frequently collapses and tends to evert. A nonexposed approach, in which the wall is inverted and closed prior to full‐thickness resection, mitigates this concern. Techniques utilizing OTSC systems or the dedicated FTRD apply this concept and substantially reduce perforation risks [37, 70, 71]. However, these methods remain fundamentally limited by lesion size.

### 3.8 Voting on Future Clinical Applications of EFTR for Epithelial Neoplasms

At the end of the session, the two moderators and seven panelists voted on the appropriateness of EFTR for epithelial neoplasms across different organs, balancing oncological risks against technical feasibility. All nine participants (100%) supported EFTR for rectal lesions, and eight (89%) supported its use for gastric lesions (Table 3).

**TABLE 3:** The results of the voting for future clinical application of EFTR for epithelial neoplasms at the end of the session in EFJ2025.

	Support	Oppose	Agreement
Esophagus	5	4	56%
Stomach	8	1	89%
Duodenum	4	5	44%
Colon	0	9	0%
Rectum	9	0	100%

Abbreviations: EFJ, endoscopic forum Japan; EFTR, endoscopic full‐thickness resection.

## 4 Discussion

We have presented an overview of EFTR for SETs. This advanced resection technique has been established as a non‐scarring approach, and several studies demonstrate its clinical efficacy and feasibility, particularly for gastric lesions. However, because secure resection and closure are essential for standardizing and generalizing this technique, further investigation is needed to establish EFTR as a mainstream minimally invasive treatment. Multidisciplinary discussions regarding extraluminal organs and surrounding anatomy are crucial for gastroenterologists to perform safe and efficient EFTR. This is particularly relevant for the esophagus, duodenum, and rectum, where anatomical considerations directly affect procedural safety. Evaluating cost‐effectiveness, risk–benefit analysis, and long‐term outcomes of EFTR compared with laparoscopic endoscopic cooperative surgery (LECS), alongside medical reimbursement assessments, represents key directions for future research in Japan.

We also discussed the clinical applications of EFTR for epithelial tumors. Clinicians must address several challenges and develop corresponding solutions to optimize this advanced technique. Oncologically, Japanese guidelines recommend surgical resection for lesions at risk of LNM [43, 72, 73, 74]. However, given Japan's aging population, surgery may constitute overtreatment for patients with limited life expectancies or significant comorbidities. Therefore, EFTR serves as an alternative minimally invasive local resection approach for select invasive cancers. Further investigation is needed to determine the clinical indications for EFTR, weighing the benefits of minimal invasiveness against the risks of metastatic recurrence and dissemination. Future multimodal artificial intelligence applications may play an important role in refining these indications.

In contrast to EMR and ESD, EFTR requires an endoscopic approach beyond the muscle layer, inherently causing GI wall perforation. This subsequent loss of insufflation yields poor operative field visualization, complicating complete resection and hemostasis. Additionally, durable and sustained closure is essential to validate the concept of a non‐scarring approach. The nonexposed approach offers a key solution to address both these concerns and the risk of peritoneal dissemination. Closed LECS, also reported as nonexposed endoscopic wall‐inversion surgery (NEWS) or the combination of laparoscopic and endoscopic approaches to neoplasia with non‐exposure technique (CLEAN‐NET), is an option for achieving nonexposed full‐thickness resection with surgical suturing [75, 76, 77]. Although its technical concepts and clinical applicability differ substantially from those of EFTR, this combined approach may be considered for lesions in areas where pre‐resection endoscopic closure currently proves technically difficult. Further innovations in devices and the refinement of resection techniques are needed to confirm this concept.

As reflected by the unanimous panel vote, EFTR offers specific advantages for select patients with rectal lesions. Given that postoperative complications occur in 25%–30% of standard rectal surgeries, even when performed laparoscopically [78, 79], EFTR presents a safe and feasible treatment option for select rectal lesions. Notably, LECS cannot be utilized for lesions below the peritoneal reflection in the lower rectum, further highlighting the clinical utility of EFTR in this anatomical region.

In conclusion, EFTR is clinically feasible for treating gastric SETs. Future investigations and technical innovations are required to expand the clinical indications for EFTR in epithelial neoplasms.

## Author Contributions

**Seiichiro Abe:** conceptualization, writing – original draft, investigation, data curation, and final approval of the article. **Kenichiro Imai:** conceptualization, writing – original draft, investigation, data curation, writing – review and editing, and final approval of the article. **Teppei Akimoto:** writing – original draft, investigation, data curation, writing – review, editing, and final approval of the article. **Takashi Kanesaka:** writing – original draft, investigation, data curation, writing – review and editing, and final approval of the article. **Yosuke Minoda:** writing – original draft, investigation, data curation, and writing – review and editing; final approval of the article. **Noriko Nishiyama:** writing – original draft, investigation, data curation, writing – review and editing, and final approval of the article. **Chiko Sato:** writing – original draft, investigation, data curation, writing – review and editing, and final approval of the article. **Akira Ouchi:** data curation, writing – review and editing, and final approval of the article. **Hon Chi Yip:** data curation, writing – review and editing, and final approval of the article. **Takao Itoi:** writing – review and editing, and final approval of the article. **Mitsuhiro Fujishiro:** writing – review and editing, and final approval of the article. **Yutaka Saito:** writing – review and editing, and final approval of the article.

## Funding

Olympus Marketing Inc. supported the open access publication of this paper.

## Ethics Statement

The authors have nothing to report.

## Conflicts of Interest

Seiichiro Abe received grants from Olympus, FUJIFILM, AI Medical, consulting fees from Olympus, honoraria from Olympus Corporation. and Olympus Marketing Inc., Boston Scientific, Takeda Pharmaceutical Company, EA Pharma CREO medical, Eisai, Otsuka Pharmaceutical Factory, Covidien, FUJIFILM, Johnson & Johnson, AI Medical, and MC Medical, leadership or fiduciary role in DEN Open, Gastrointestinal Endoscopy, Digestion, and Clinical Endoscopy. Kenichiro Imai received grants from KANEKA Corporation, the Japanese Foundation for research and promotion of endoscopy, the Japanese Gastroenterological Association, consulting fee from Olympus Corporation and Olympus Marketing Inc., and Boston Scientific, honoraria from Olympus Corporation. and Olympus Marketing Inc., Boston Scientific, Takeda Pharmaceutical Company, EA Pharma, 3D matrix, KANEKA Corporation, Kaigen‐pharma, TOP Corporation, support for attending meetings from Boston Scientific, leadership or fiduciary role in Asian Novel Bio‐Imaging and Intervention Group, Asian Endoscopy Research Forum, Endoscopy, Digestive Endoscopy, and equipment from Olympus and 3D Matrix. Teppei Akimoto received grants from Olympus Corporation., Consulting fees from Olympus Corporation and Medtronic plc, honoraria from Olympus Corporation., Olympus Marketing Inc., Boston Scientific Corporation, Support for attending meetings from Olympus Corporation., leadership or fiduciary role in Gastroenterological Endoscopy Society, and equipment from AI Medical Service Inc. Takashi Kanesaka received grants from Japan Gastroenterological Endoscopy Society and Osaka Foundation for the Prevention of Cancer and Cardiovascular Diseases, honoraria from Olympus Corporation. and Olympus Marketing Inc. Yosuke Minoda received grants from Olympus Medical systems and MC medical, and honoraria from Olympus Corporation. and Olympus Marketing Inc. Noriko Nishiyama received honoraria from Olympus Corporation. and Olympus Marketing Inc. Chiko Sato received honoraria from Olympus Corporation. and Olympus Marketing Inc. Akira Ouchi received honoraria from Olympus Corporation. and Olympus Marketing Inc., and Medtronic Thailand. Hon Chi Yip received grants from Olympus Medical Corporations, honoraria from Olympus Corporation and Olympus Marketing Inc., and stock in EndoR Surgical Limited. Takao Itoi received honoraria from Olympus Corporation. and Olympus Marketing Inc. Mitsuhiro Fujishiro received honoraria from Olympus Corporation and Olympus Marketing Inc. Yutaka Saito received honoraria from Olympus Corporation and Olympus Marketing Inc.

## References

[1] M. Y. Cai , F. Martin Carreras‐Presas , and P. H. Zhou , “Endoscopic Full‐Thickness Resection for Gastrointestinal Submucosal Tumors,” Digestive Endoscopy 30, no. Suppl 1 (2018): 17–24, 10.1111/den.13003. 29658639

[2] L. W. Zwager , B. A. J. Bastiaansen , M. E. S. Bronzwaer , et al., “Endoscopic Full‐Thickness Resection (eFTR) of Colorectal Lesions: Results From the Dutch Colorectal eFTR Registry,” Endoscopy 52, no. 11 (2020): 1014–1023, 10.1055/a-1176-1107. 32498100

[3] J. Chen , F. Wang , Y. Wang , et al., “A Comparison of Postoperative Outcomes Between Robotic‐Assisted and Laparoscopic‐Assisted Total Gastrectomy: A Comprehensive Meta‐Analysis and Systematic Review,” BMC Surgery 25, no. 1 (2025): 212, 10.1186/s12893-025-02934-5. 40375289 PMC12079958

[4] Q. Feng , W. Yuan , T. Li , et al., “Robotic Versus Laparoscopic Surgery for Middle and Low Rectal Cancer (Real): Short‐Term Outcomes of a Multicentre Randomised Controlled Trial,” Lancet Gastroenterology & Hepatology 7, no. 11 (2022): 991–1004, 10.1016/s2468-1253(22)00248-5. 36087608

[5] N. Hasuike , H. Ono , N. Boku , et al., “A Non‐Randomized Confirmatory Trial of an Expanded Indication for Endoscopic Submucosal Dissection for Intestinal‐Type Gastric Cancer (Ct1a): The Japan Clinical Oncology Group Study (Jcog0607),” Gastric Cancer 21, no. 1 (2018): 114–123, 10.1007/s10120-017-0704-y. 28224238

[6] K. Takizawa , H. Ono , N. Hasuike , et al., “A Nonrandomized, Single‐Arm Confirmatory Trial of Expanded Endoscopic Submucosal Dissection Indication for Undifferentiated Early Gastric Cancer: Japan Clinical Oncology Group Study (Jcog1009/1010),” Gastric Cancer 24, no. 2 (2021): 479–491, 10.1007/s10120-020-01134-9. 33161444

[7] H. R. Aslanian , A. Sethi , M. S. Bhutani , et al., “Asge Guideline for Endoscopic Full‐Thickness Resection and Submucosal Tunnel Endoscopic Resection,” VideoGIE 4, no. 8 (2019): 343–350, 10.1016/j.vgie.2019.03.010. 31388606 PMC6669323

[8] Y. Shimamura , H. Inoue , K. Yamamoto , K. Owada , and I. Tanaka , “Advancements in Minimally Invasive Endoscopic Treatment: Navigating Deeper Layers for Upper Gastrointestinal Lesion,” Digestive Endoscopy 36, no. 10 (2024): 1094–1104, 10.1111/den.14828. 38867345

[9] L. S. D'Souza , D. Yang , and D. Diehl , “Aga Clinical Practice Update on Endoscopic Full‐Thickness Resection for the Management of Gastrointestinal Subepithelial Lesions: Commentary,” Gastroenterology 166, no. 2 (2024): 345–349, 10.1053/j.gastro.2023.11.016. 38108671

[10] B. R. Liu , J. T. Song , B. Qu , J. F. Wen , J. B. Yin , and W. Liu , “Endoscopic Muscularis Dissection for Upper Gastrointestinal Subepithelial Tumors Originating From the Muscularis Propria,” Surgical Endoscopy 26, no. 11 (2012): 3141–3148, 10.1007/s00464-012-2305-5. 22580875

[11] F. Liu , S. Zhang , W. Ren , et al., “The Fourth Space Surgery: Endoscopic Subserosal Dissection for Upper Gastrointestinal Subepithelial Tumors Originating From the Muscularis Propria Layer,” Surgical Endoscopy 32, no. 5 (2018): 2575–2582, 10.1007/s00464-017-5985-z. 29264757

[12] I. Tanaka , Y. Shimamura , H. Inoue , et al., “Endoscopic Resection for Gastric Submucosal Tumors: A Single‐Center Experience in Japan,” DEN Open 5, no. 1 (2025): e402, 10.1002/deo2.402. 39011512 PMC11249007

[13] T. Toyonaga , Y. Ohara , S. Baba , et al., “Peranal Endoscopic Myectomy (PAEM) for Rectal Lesions With Severe Fibrosis and Exhibiting the Muscle‐Retracting Sign,” Endoscopy 50, no. 8 (2018): 813–817, 10.1055/a-0602-3905. 29883977

[14] L. M. G. Moons , B. A. J. Bastiaansen , M. C. Richir , et al., “Endoscopic Intermuscular Dissection for Deep Submucosal Invasive Cancer in the Rectum: A New Endoscopic Approach,” Endoscopy 54, no. 10 (2022): 993–998, 10.1055/a-1748-8573. 35073588

[15] H. Suzuki and K. Ikeda , “Endoscopic Mucosal Resection and Full Thickness Resection With Complete Defect Closure for Early Gastrointestinal Malignancies,” Endoscopy 33, no. 5.0 (2001): 437–439, 10.1055/s-2001-14269. 11396763

[16] P. H. Zhou , M. Y. Cai , L. Q. Yao , et al., “Peroral Endoscopic Myotomy for Esophageal Achalasia: Report of 42 Cases,” Zhonghua Wei Chang Wai Ke Za Zhi 14, no. 9 (2011): 705–708. 21948538

[17] S. Shichijo , N. Uedo , A. Sawada , et al., “Endoscopic Full‐Thickness Resection for Gastric Submucosal Tumors: Japanese Multicenter Prospective Study,” Digestive Endoscopy 36, no. 7 (2024): 811–821, 10.1111/den.14717. 37914400

[18] Y. Zhu , M. D. Xu , C. Xu , et al., “Microscopic Positive Tumor Margin Does Not Increase the Rate of Recurrence in Endoscopic Resected Gastric Mesenchymal Tumors Compared to Negative Tumor Margin,” Surgical Endoscopy 34, no. 1.0 (2020): 159–169, 10.1007/s00464-019-06744-8. 31139992

[19] S. Hirota , U. Tateishi , Y. Nakamoto , et al., “English Version of Japanese Clinical Practice Guidelines 2022 for Gastrointestinal Stromal Tumor (GIST) Issued by the Japan Society of Clinical Oncology,” International Journal of Clinical Oncology 29, no. 6.0 (2024): 647–680, 10.1007/s10147-024-02488-1. 38609732 PMC11130037

[20] P. G. Casali , J. Y. Blay , N. Abecassis , et al., “Gastrointestinal Stromal Tumours: Esmo‐Euracan‐Genturis Clinical Practice Guidelines for Diagnosis, Treatment and Follow‐Up,” Annals of Oncology 33, no. 1.0 (2022): 20–33, 10.1016/j.annonc.2021.09.005. 34560242

[21] T. Chen , Y. W. Zhang , J. J. Lian , et al., “No‐Touch Endoscopic Full‐Thickness Resection Technique for Gastric Gastrointestinal Stromal Tumors,” Endoscopy 55, no. 6.0 (2023): 557–562, 10.1055/a-2013-1902. 36758584

[22] I. Dalal and I. Andalib , “Advances in Endoscopic Resection: A Review of Endoscopic Submucosal Dissection (ESD), Endoscopic Full Thickness Resection (EFTR) and Submucosal Tunneling Endoscopic Resection (STER),” Translational Gastroenterology and Hepatology 7 (2022): 19, 10.21037/tgh-2020-10. 35548477 PMC9081920

[23] N. Eleftheriadis , H. Inoue , H. Ikeda , M. Onimaru , R. Maselli , and G. Santi , “Submucosal Tunnel Endoscopy: Peroral Endoscopic Myotomy and Peroral Endoscopic Tumor Resection,” World Journal of Gastrointestinal Endoscopy 8, no. 2 (2016): 86–103, 10.4253/wjge.v8.i2.86. 26839649 PMC4724034

[24] M. Onimaru , H. Inoue , R. Bechara , et al., “Clinical Outcomes of Per‐Oral Endoscopic Tumor Resection for Submucosal Tumors in the Esophagus and Gastric Cardia,” Digestive Endoscopy 32, no. 3 (2020): 328–336, 10.1111/den.13471. 31234231

[25] T. Chen , P. H. Zhou , Y. Chu , et al., “Long‐Term Outcomes of Submucosal Tunneling Endoscopic Resection for Upper Gastrointestinal Submucosal Tumors,” Annals of Surgery 265, no. 2.0 (2017): 363–369, 10.1097/SLA.0000000000001650. 28059965

[26] A. Granata , A. Martino , D. Ligresti , F. Tuzzolino , G. Lombardi , and M. Traina , “Exposed Endoscopic Full‐Thickness Resection Without Laparoscopic Assistance for Gastric Submucosal Tumors: A Systematic Review and Pooled Analysis,” Digestive and Liver Disease 54, no. 6.0 (2022): 729–736, 10.1016/j.dld.2021.09.014. 34654680

[27] N. Tada , H. Kobara , N. Nishiyama , S. Fujihara , T. Masaki , and N. Uedo , “Current Status of Endoscopic Full‐Thickness Resection for Gastric Subepithelial Tumors: A Literature Review Over Two Decades,” Digestion 104, no. 6.0 (2023): 415–429, 10.1159/000530679. 37423206

[28] D. Shi , R. Li , W. Chen , et al., “Application of Novel Endoloops to Close the Defects Resulted From Endoscopic Full‐Thickness Resection With Single‐Channel Gastroscope: A Multicenter Study,” Surgical Endoscopy 31, no. 2 (2017): 837–842, 10.1007/s00464-016-5041-4. 27351654

[29] J. Guo , Z. Liu , S. Sun , et al., “Endoscopic Full‐Thickness Resection With Defect Closure Using an Over‐The‐Scope Clip for Gastric Subepithelial Tumors Originating From the Muscularis Propria,” Surgical Endoscopy 29, no. 11 (2015): 3356–3362, 10.1007/s00464-015-4076-2. 25701060 PMC4607707

[30] T. Nomura , S. Sugimoto , H. Nakamura , et al., “Reopenable Clip Over Line Method for the Closure of Full‐Thickness Defect After Gastric Endoscopic Full‐Thickness Resection,” Endoscopy 54, no. S 02 (2022): E808–E809, 10.1055/a-1824-4919. 35523218 PMC9735295

[31] S. Shichijo , N. Uedo , H. Mori , et al., “Reopenable clip over‐the‐line method in endoscopic full‐thickness resection of gastric submucosal tumors: A historical control study,” DEN Open 5, no. 1 (2025): e70067, 10.1002/deo2.70067. 39882504 PMC11774660

[32] C. Yu , G. Liao , C. Fan , et al., “Long‐Term Outcomes of Endoscopic Resection of Gastric Gists,” Surgical Endoscopy 31, no. 11 (2017): 4799–4804, 10.1007/s00464-017-5557-2. 28424911

[33] Y. Zhang , X. L. Mao , X. B. Zhou , et al., “Long‐Term Outcomes of Endoscopic Resection for Small (≤ 4.0 cm) Gastric Gastrointestinal Stromal Tumors Originating From the Muscularis Propria Layer,” World Journal of Gastroenterology 24, no. 27 (2018): 3030–3037, 10.3748/wjg.v24.i27.3030. 30038470 PMC6054947

[34] J. Gao , Z. Liu , X. Liu , et al., “Follow‐Up Analysis and Research of Very Low‐Risk and Low‐Risk Gastrointestinal Stromal Tumors After Endoscopic Resection,” Scientific Reports 14, no. 1 (2024): 17872, 10.1038/s41598-024-68460-1. 39090269 PMC11294471

[35] Y. Wei , Q. Zhou , M. Ji , S. Zhang , and P. Li , “Over‐The‐Scope Clip‐Assisted Endoscopic Full‐Thickness Resection Has Potential to Treat Complex Nonampullary Duodenal Lesions: A Single‐Center Case Series,” BMC Gastroenterology 21, no. 1.0 (2021): 476, 10.1186/s12876-021-02068-x. 34911448 PMC8675504

[36] A. Wannhoff , Z. Nabi , L. M. G. Moons , et al., “International, Multicenter Analysis of Endoscopic Full‐Thickness Resection of Duodenal Neuroendocrine Tumors,” American Journal of Gastroenterology 120, no. 12 (2025): 2800–2809, 10.14309/ajg.0000000000003409. 40079474

[37] A. Schmidt , T. Beyna , B. Schumacher , et al., “Colonoscopic Full‐Thickness Resection Using an Over‐the‐Scope Device: A Prospective Multicentre Study in Various Indications,” Gut 67, no. 7.0 (2018): 1280–1289, 10.1136/gutjnl-2016-313677. 28798042

[38] J. Mueller , A. Kuellmer , M. Schiemer , R. Thimme , and A. Schmidt , “Current Status of Endoscopic Full‐Thickness Resection With the Full‐Thickness Resection Device,” Digestive Endoscopy 35, no. 2.0 (2023): 232–242, 10.1111/den.14425. 35997598

[39] P. H. Zhou , L. Q. Yao , X. Y. Qin , et al., “Endoscopic Full‐Thickness Resection Without Laparoscopic Assistance for Gastric Submucosal Tumors Originated From the Muscularis Propria,” Surgical Endoscopy 25 (2011): 2926–2931, 10.1007/s00464-011-1644-y. 21424195

[40] B. Meier , B. Stritzke , A. Kuellmer , et al., “Efficacy and Safety of Endoscopic Full‐Thickness Resection in the Colorectum: Results From the German Colonic FTRD Registry,” American Journal of Gastroenterology 115 (2020): 1998–2006, 10.14309/ajg.0000000000000795. 32833733

[41] T. Eguchi , Y. Nakanishi , T. Shimoda , et al., “Histopathological Criteria for Additional Treatment After Endoscopic Mucosal Resection for Esophageal Cancer: Analysis of 464 Surgically Resected Cases,” Modern Pathology 19, no. 3 (2006): 475–480, 10.1038/modpathol.3800557. 16444191

[42] W. Hatta , T. Koike , K. Uno , N. Asano , and A. Masamune , “Management of Superficial Esophageal Squamous Cell Carcinoma and Early Gastric Cancer Following Non‐Curative Endoscopic Resection,” Cancers 14, no. 15 (2022): 3757, 10.3390/cancers14153757. 35954421 PMC9367302

[43] Japanese Gastric Cancer Association , “Japanese Gastric Cancer Treatment Guidelines 2021 (6th Edition),” Gastric Cancer 26, no. 1 (2023): 1–25, 10.1007/s10120-022-01331-8. 36342574 PMC9813208

[44] W. Hatta , T. Gotoda , T. Oyama , et al., “A Scoring System to Stratify Curability After Endoscopic Submucosal Dissection for Early Gastric Cancer: ‘Ecura System’,” American Journal of Gastroenterology 112, no. 6 (2017): 874–881, 10.1038/ajg.2017.95. 28397873

[45] N. Hanaoka , S. Tanabe , T. Mikami , I. Okayasu , and K. Saigenji , “Mixed‐Histologic‐Type Submucosal Invasive Gastric Cancer as a Risk Factor for Lymph Node Metastasis: Feasibility of Endoscopic Submucosal Dissection,” Endoscopy 41, no. 5 (2009): 427–432, 10.1055/s-0029-1214495. 19418397

[46] T. Hirasawa , T. Gotoda , S. Miyata , et al., “Incidence of Lymph Node Metastasis and the Feasibility of Endoscopic Resection for Undifferentiated‐Type Early Gastric Cancer,” Gastric Cancer 12, no. 3 (2009): 148–152, 10.1007/s10120-009-0515-x. 19890694

[47] M. Bauder , A. Schmidt , and K. Caca , “Endoscopic Full‐Thickness Resection of Duodenal Lesions‐a Retrospective Analysis of 20 Ftrd Cases,” United European Gastroenterology Journal 6, no. 7 (2018): 1015–1021, 10.1177/2050640618773517. 30228889 PMC6137579

[48] K. Nakagawa , M. Sho , K. I. Okada , et al., “Surgical Results of Non‐Ampullary Duodenal Cancer: A Nationwide Survey in Japan,” Journal of Gastroenterology 57, no. 2 (2022): 70–81, 10.1007/s00535-021-01841-9. 34988688

[49] S. Yoshimizu , H. Kawachi , Y. Yamamoto , et al., “Clinicopathological Features and Risk Factors for Lymph Node Metastasis in Early‐Stage Non‐Ampullary Duodenal Adenocarcinoma,” Journal of Gastroenterology 55, no. 8 (2020): 754–762, 10.1007/s00535-020-01696-6. 32533301

[50] T. Ito , T. Masui , I. Komoto , et al., “JNETS Clinical Practice Guidelines for Gastroenteropancreatic Neuroendocrine Neoplasms: Diagnosis, Treatment, and Follow‐Up: A Synopsis,” Journal of Gastroenterology 56, no. 11 (2021): 1033–1044, 10.1007/s00535-021-01827-7. 34586495 PMC8531106

[51] Y. Hashiguchi , K. Muro , Y. Saito , et al., “Japanese Society for Cancer of the Colon and Rectum (JSCCR) Guidelines 2019 for the Treatment of Colorectal Cancer,” International Journal of Clinical Oncology 25, no. 1 (2020): 1–42, 10.1007/s10147-019-01485-z. 31203527 PMC6946738

[52] K. Nakadoi , S. Tanaka , H. Kanao , et al., “Management of T1 Colorectal Carcinoma With Special Reference to Criteria for Curative Endoscopic Resection,” Journal of Gastroenterology and Hepatology 27, no. 6 (2012): 1057–1062, 10.1111/j.1440-1746.2011.07041.x. 22142484

[53] H. Tanaka , K. Yamashita , Y. Urabe , T. Kuwai , and S. Oka , “Management of T1 Colorectal Cancer,” Digestion 106, no. 2 (2025): 122–130, 10.1159/000540594. 39097960

[54] E. Rajan and L. M. Wong Kee Song , “Endoscopic Full Thickness Resection,” Gastroenterology 154, no. 7 (2018): 1925–1937.e2, 10.1053/j.gastro.2018.02.020. 29486198

[55] H. Kitakata , T. Itoh , S. Kinami , et al., “Sealed Endoscopic Full‐Thickness Resection for Gastric Cancer: A Pilot Study in an Ex Vivo and In Vivo Porcine Model,” Endoscopy International Open 7, no. 1 (2019): E36–e42, 10.1055/a-0777-1954. 30648137 PMC6327734

[56] H. Mori , N. Uedo , S. Shichijo , et al., “Endoscopic Full‐Thickness Resection for Gastric Submucosal Tumor: A Technical Analysis Study (With Video),” DEN Open 6, no. 1 (2026): e70198, 10.1002/deo2.70198. 40933880 PMC12417311

[57] M. Abdallah , G. Suryawanshi , N. McDonald , et al., “Endoscopic Full‐Thickness Resection for Upper Gastrointestinal Tract Lesions: A Systematic Review and Meta‐Analysis,” Surgical Endoscopy 37, no. 5 (2023): 3293–3305, 10.1007/s00464-022-09801-x. 36517704

[58] B. H. Song , J. Bahetinuer , Y. S. Zhong , H. C. Yip , P. H. Zhou , and M. Y. Cai , “Endoscopic Full‐Thickness Resection for the Treatment of Gastric Gastrointestinal Stromal Tumors,” Clinical Endoscopy 59 (2025): 9–20, 10.5946/ce.2025.001. 40899241 PMC12933609

[59] K. Wang , P. Gao , M. Cai , B. Song , and P. Zhou , “Endoscopic Full‐Thickness Resection, Indication, Methods and Perspectives,” Digestive Endoscopy 35, no. 2 (2023): 195–205, 10.1111/den.14474. 36355358

[60] K. Higuchi , O. Goto , E. Koizumi , et al., “Potential for Expanded Application of Endoscopic Hand Suturing: A Pilot Study of 15 Cases,” Endoscopy International Open 12, no. 4 (2024): E507–E512, 10.1055/a-2284-9492. 38585020 PMC10997422

[61] O. Goto , M. Sasaki , H. Ishii , et al., “A New Endoscopic Closure Method for Gastric Mucosal Defects: Feasibility of Endoscopic Hand Suturing in an Ex Vivo Porcine Model (With Video),” Endoscopy International Open 2, no. 2 (2014): E111–E116, 10.1055/s-0034-1377180. 26135255 PMC4424863

[62] T. Matsui , H. Kobara , N. Nishiyama , et al., “Comparison of Purse‐String Suture Versus Over‐the‐Scope Clip for Gastric Endoscopic Full‐Thickness Closure: Traction and Leak Pressure Testing in Ex Vivo Porcine Model,” BMC Surgery 23, no. 1 (2023): 20, 10.1186/s12893-023-01920-z. 36703127 PMC9878917

[63] A. Granata , A. Martino , D. Ligresti , et al., “Closure Techniques in Exposed Endoscopic Full‐Thickness Resection: Overview and Future Perspectives in the Endoscopic Suturing Era,” World Journal of Gastrointestinal Surgery 13, no. 7 (2021): 645–654, 10.4240/wjgs.v13.i7.645. 34354798 PMC8316845

[64] N. Yahagi , T. Nishizawa , T. Akimoto , Y. Ochiai , and O. Goto , “New Endoscopic Suturing Method: String Clip Suturing Method,” Gastrointestinal Endoscopy 84, no. 6 (2016): 1064–1065, 10.1016/j.gie.2016.05.054. 27327846

[65] T. Nishizawa , T. Akimoto , T. Uraoka , et al., “Endoscopic String Clip Suturing Method: A Prospective Pilot Study (With Video),” Gastrointestinal Endoscopy 87, no. 4 (2018): 1074–1078, 10.1016/j.gie.2017.11.007. 29154910

[66] Y. Yamasaki , M. Ohmori , J. Toyosawa , S. Ako , and H. Okada , “Modified Line‐Assisted Complete Closure of the Defect After Gastric Endoscopic Full‐Thickness Resection: A Pilot Study in Porcine Models,” Endoscopy International Open 10, no. 5 (2022): E609–E615, 10.1055/a-1785-8589. 35571472 PMC9106438

[67] H. Kobara , N. Nishiyama , S. Fujihara , et al., “Traction‐Assisted Endoscopic Full‐Thickness Resection Followed by O‐Ring and Over‐The‐Scope Clip Closure in the Stomach: An Animal Experimental Study,” Endoscopy International Open 9, no. 1 (2021): E51–E57, 10.1055/a-1287-7482. 33403236 PMC7775815

[68] N. Nishiyama , S. Fujihara , N. Tada , et al., “Efficacy and Cost‐Effectiveness of a Novel Dual Gasping Forceps‐Assisted Over‐The‐Scope Clip Inverted Closure After Gastric Endoscopic Full‐Thickness Resection,” Endoscopy 55, no. S 01 (2023): E870–E871, 10.1055/a-2108-1037. 37433319 PMC10335865

[69] H. Mori , H. Kobara , S. Fujihara , et al., “Feasibility of Pure EFTR Using an Innovative New Endoscopic Suturing Device: The Double‐Arm‐Bar Suturing System (With Video),” Surgical Endoscopy 28, no. 2 (2014): 683–690, 10.1007/s00464-013-3266-z. 24202707

[70] T. Tashima , S. Ryozawa , Y. Tanisaka , et al., “Endoscopic Resection Using an Over‐The‐Scope Clip for Duodenal Neuroendocrine Tumors,” Endoscopy International Open 9, no. 5 (2021): E659–E666, 10.1055/a-1374-6141. 33937505 PMC8062228

[71] Y. Ogata , W. Hatta , and A. Masamune , “Endoscopic Muscularis Dissection With Over‐The‐Scope Clip: Novel Resection Technique for Duodenal Neuroendocrine Tumors,” Digestive Endoscopy 36, no. 8 (2024): 955–956, 10.1111/den.14833. 38804154

[72] H. Ono , K. Yao , M. Fujishiro , et al., “Guidelines for Endoscopic Submucosal Dissection and Endoscopic Mucosal Resection for Early Gastric Cancer (Second Edition),” Digestive Endoscopy 33, no. 1 (2021): 4–20, 10.1111/den.13883. 33107115

[73] R. Ishihara , M. Arima , T. Iizuka , et al., “Endoscopic Submucosal Dissection/Endoscopic Mucosal Resection Guidelines for Esophageal Cancer,” Digestive Endoscopy 32, no. 4 (2020): 452–493, 10.1111/den.13654. 32072683

[74] S. Tanaka , H. Kashida , Y. Saito , et al., “Japan Gastroenterological Endoscopy Society Guidelines for Colorectal Endoscopic Submucosal Dissection/Endoscopic Mucosal Resection,” Digestive Endoscopy 32, no. 2 (2020): 219–239, 10.1111/den.13545. 31566804

[75] M. Onimaru , H. Inoue , H. Ikeda , et al., “Combination of Laparoscopic and Endoscopic Approaches for Neoplasia With Non‐Exposure Technique (Clean‐Net) for Gastric Submucosal Tumors: Updated Advantages and Limitations,” Annals of Translational Medicine 7, no. 20 (2019): 582, 10.21037/atm.2019.09.19. 31807563 PMC6861760

[76] S. Kikuchi , M. Nishizaki , S. Kuroda , et al., “Nonexposure Laparoscopic and Endoscopic Cooperative Surgery (Closed Laparoscopic and Endoscopic Cooperative Surgery) for Gastric Submucosal Tumor,” Gastric Cancer 20, no. 3 (2017): 553–557, 10.1007/s10120-016-0641-1. 27599829

[77] O. Goto , H. Takeuchi , M. Sasaki , et al., “Laparoscopy‐Assisted Endoscopic Full‐Thickness Resection of Gastric Subepithelial Tumors Using a Nonexposure Technique,” Endoscopy 48, no. 11 (2016): 1010–1015, 10.1055/s-0042-111000. 27448050

[78] M. Yasui , I. Takemasa , Y. Miyake , et al., “Tumor Size as an Independent Risk Factor for Postoperative Complications in Laparoscopic Low Anterior Resection for Advanced Rectal Cancer: A Multicenter Japanese Study,” Surgical Laparoscopy, Endoscopy & Percutaneous Techniques 27 (2017): 98–103, 10.1097/SLE.0000000000000377. 28141746

[79] K. Hida , R. Okamura , Y. Sakai , et al., “Open Versus Laparoscopic Surgery for Advanced Low Rectal Cancer: A Large, Multicenter, Propensity Score Matched Cohort Study in Japan,” Annals of Surgery 268, no. 2 (2018): 318–324, 10.1097/sla.0000000000002329. 28628565 PMC6092102

